# Retroperitoneal kaposiform hemangioendothelioma with kasabach-merritt phenomenon in children: A case report and review of the literature

**DOI:** 10.3389/fped.2023.1138689

**Published:** 2023-03-17

**Authors:** Junming Huo, Song Chen, Jing Li, Chengjun Liu

**Affiliations:** Department of Critical Care Medicine, National Clinical Research Center for Child Health and Disorders, Ministry of Education Key Laboratory of Child Development and Disorders, China International Science and Technology Cooperation Base of Child Development and Critical Disorders, Chongqing Key Laboratory of Pediatrics, Children’s Hospital of Chongqing Medical University, Chongqing, China

**Keywords:** kaposiform hemangioendothelioma, retroperitoneal, kasabach-Merritt phenomenon, pediatric, literature review

## Abstract

**Objective:**

To investigate the clinical features, diagnosis and treatment methods and prognosis of retroperitoneal Kaposiform hemangioendothelioma (R-KHE) in children.

**Methods:**

The clinical data of an infant with R-KHE was retrospectively analyzed. Literature on R-KHE in pediatrics were retrieved in databases including Wanfang, CNKI and PubMed as of April 2022.

**Results:**

A 1 month and 6 days female infant with R-KHE was reported. After the diagnosis was confirmed by biopsy and pathological examination, the patient was treated by interventional embolization, and a combined therapy with glucocorticoid, vincristine, sirolimus and propranolol. The patient has been followed up for 1 year and 2 months, and is still alive with tumor. Through literature search, a total of 15 children, together with the case in our report, were included. The main manifestations were diversity among those patients. 14 cases have combined Kasabach-Merritt phenomenon (KMP). 6 cases accepted surgery plus drug therapy. 4 cases accepted only surgery, and 4 cases only accepted drug therapy. While drug therapy plus radiotherapy were employed to 1 case. Improvement was observed in 11 cases, with significantly reduced tumor and survival with tumor. Tumor disappeared completely in 2 cases. While 2 cases suffered death.

**Conclusion:**

R-KHE has diverse clinical presentations and non-specificity in symptoms and imaging examinations, and most cases accompanied with KMP. Methods for R-KHE treatment include surgical resection, interventional embolization and drug therapy. Close attention needs to be paid to the adverse reactions of the drug during the course of treatment.

## 1. Introduction

Kaposiform hemangioendothelioma (KHE) is a rare vascular tumor with an estimated incidence of 0.91/100,000 (1). It often involves the dermis and extends to the subcutaneous tissue. It is characterized by moderate malignancy and local aggression, and the risk of death in KHE patients is often associated with the Kasabach-Merritt phenomenon (KMP), which occurs in 42%–71% of KHE cases (2–5). KMP is characterized by profound thrombocytopenia, microangiopathic anemia and hypofibrinogenemia. Compared with superficial lesions, Retroperitoneal KHE(R-KHE) has a higher incidence of KMP (2), which means that patients with R-KHE will have a higher mortality rate. It is difficult to diagnose R-KHE, and missed diagnosis or misdiagnosis is inevitable since it has diverse clinical presentations and nonspecific symptoms, and shows no specific manifestations in routine auxiliary examinations, and most of the patients cannot tolerate surgical biopsy due to their critical condition. In addition, the optimal treatment for KHE is to be determined though there are various drugs, including glucocorticoids, vincristine (VCR), interferon alfa and sirolimus, either alone or in combination, being used. Given the challenges in diagnosis and treatment of R-KHE, we report a 1-month-old girl with R-KHE, with a review of the literature on R-KHE, in the hope of providing relevant experience in diagnosis and treatment for future clinical work.

## 2. Materials and methods

### 2.1. Clinical data

**Figure 1:**
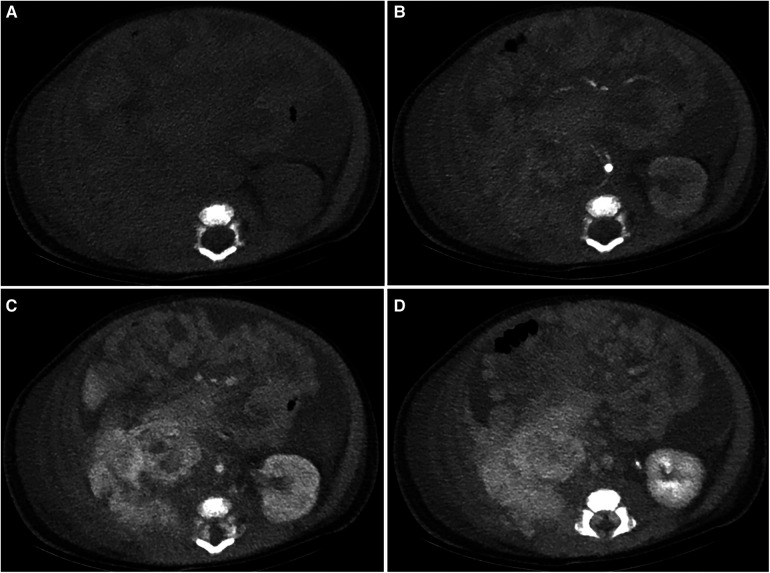
Abdominal CT results of this patient at the first visit. Plain scan (**A**) indicated a large amount of fluid-density shadow in the abdominal cavity, blurred right-sided retroperitoneal perirenal layers with an iso-low density change. Contrast enhanced scan [(**B**) arterial phase, (**C**) venous phase, (**D**) delayed phase] indicated there was inhomogeneous enhancement in the perirenal lesions.

**Figure 2:**
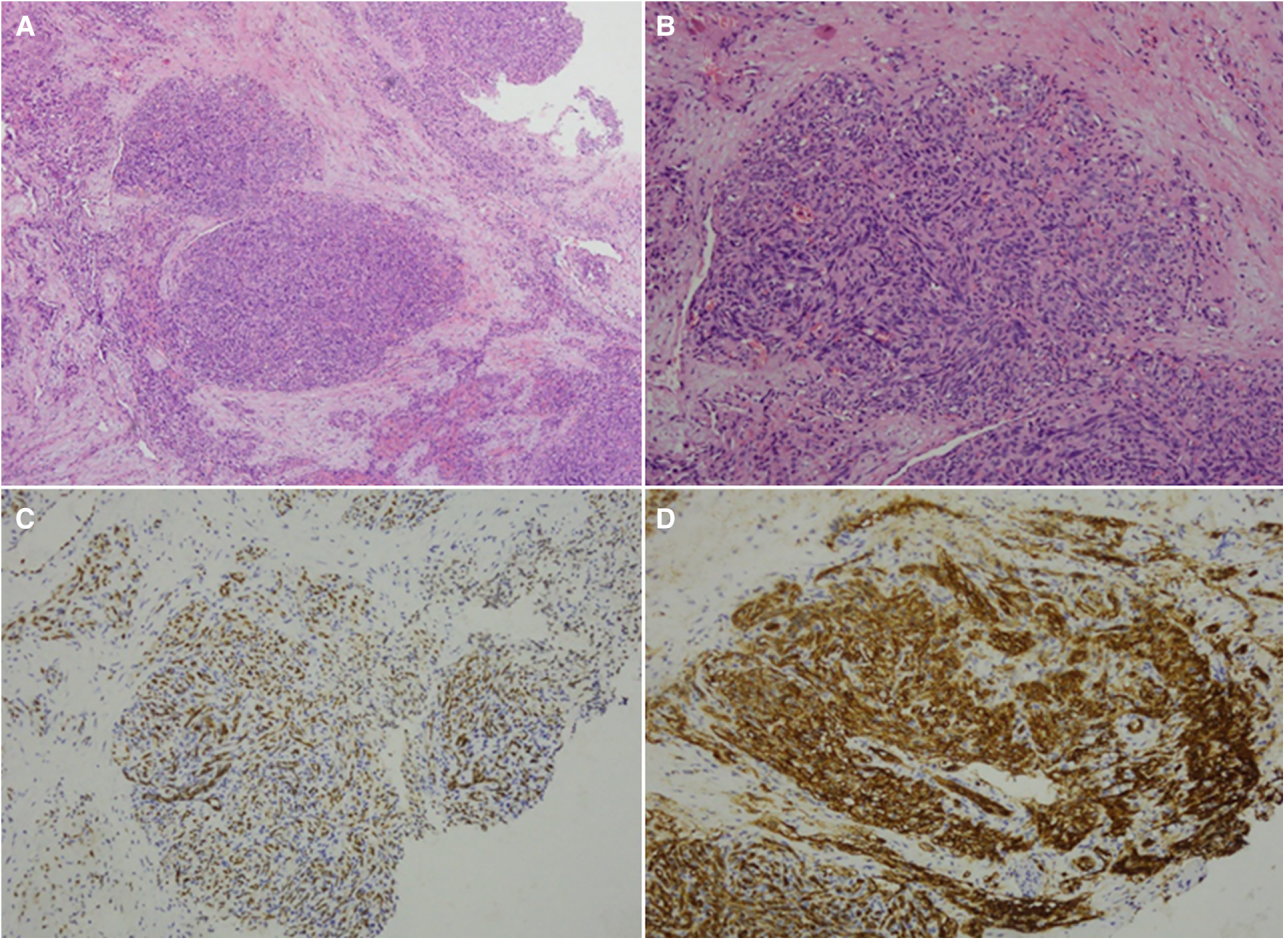
Biopsy results of the patient. Biopsy showed multifocal proliferation of cleft-like hyperplasia of small blood vessels and densely arranged spindle-shaped epithelioid cells, with red blood cells seen in the intercellular clefts [(**A**) hematoxylin and eosin, ×40; (**B**) hematoxylin and eosin, ×100]. Immunohistochemistry: FLI-1(+) [(**C)**, ×100], CD31(+) [(**D**), ×100], CD34 (+), D2-40 partially (+), REG (+), Glut-1 (-), HHV8 (-), Ki67 hotspot 40% (+). The biopsy indicated (retroperitoneal) KHE.

**Figure 3:**
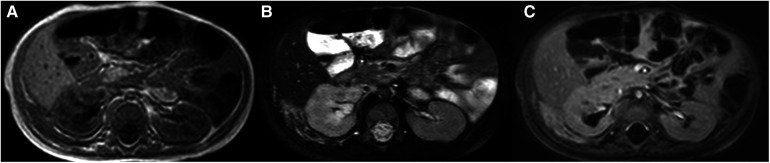
Re-examination by abdominal MRI after 10 months of treatment. The images showed that the lesion of the right kidney was significantly reduced, increased T2 signals, and there was enhancement when with contrast [(**A**) T1WI, (**B**) T2WI, (**C**) contrast-enhanced T2WI].

**Figure 4:**
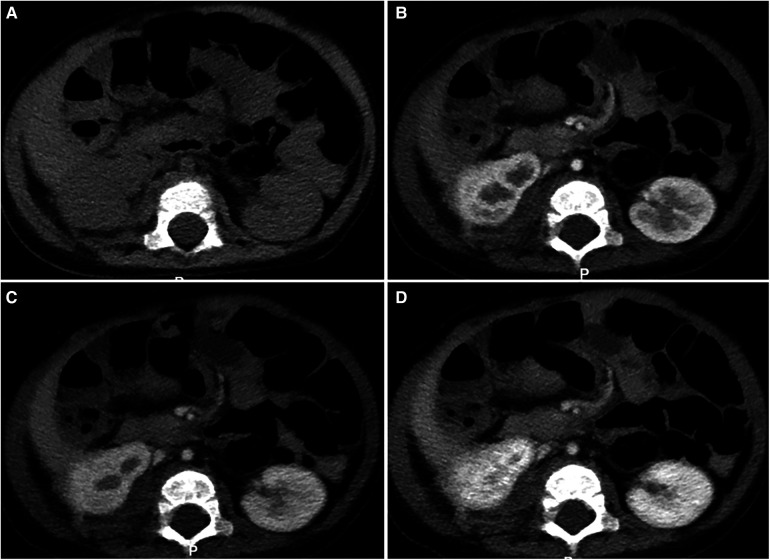
Abdominal CT results of this patient after 11 + months of treatment. The images [(**A**) plain scan, (**B**) arterial phase, (**C**) venous phase, (**D**) delayed phase] indicated the original peri-renal lesions were significantly reduced, the contour of the right kidney tended to be normal with a few residual lesions, and a little enhancement was seen with contrast.

The patient was a 1 month and 6 days female infant who was admitted to the hospital on March 6, 2021 with “abdominal distension for 20 days, skin petechiae and ecchymoses for 14 days and aggravation for 2 days”. The patient had progressive abdominal distension. Physical examination on admission revealed that the patient had pale complexion and lips, slightly swollen face, trunk and perineum, and scattered petechiae and ecchymoses all over the body, especially on the right waist. As to the auxiliary examinations, the findings of abdominal color Doppler ultrasound on March 7, 2021 included: (1) slightly enlarged liver, but no obvious abnormality in its internal structure; (2) immobile biliary sludge in the gallbladder; and (3) a medium-large amount of peritoneal effusion with cloudy components. Blood test at admission showed: WBC 8.48X10^9^/L, RBC 1.52 × 10^12^/L, Hb 53 g/L, PLT 5X10^9^/L, and coagulation factors ATTP > 150 s, PT > 150 s, fibrinogen 0.5 g/L, FDP 62.6 mg/L, and D-dimer 12.5 ng/L. CT scan of abdomen on March 11, 2021 showed in Figure 1. Treatment: anti-infection; hemostasis; repeated infusion of platelets, fresh frozen plasma (FFP), cryoprecipitate and red blood cell suspension (RBCS); invasive mechanical ventilation; methylprednisolone + gamma globulin + plasma exchange. On March 12, the patient underwent exploratory laparotomy, retroperitoneal hematoma removal, tissue biopsy and abdominal drainage. Findings during these operations included: dark red hematoma was seen from the right retroperitoneum up to the perirenal space and down to the ileocecal junction in the right lower abdomen; hemorrhage in the mesentery of the colon and the small intestine, with obvious swelling; canal stenosis due to compression by right colon hematoma; and 300 ml blood lose during the operation without obvious bleeding site found. The pathological findings of the biopsy on March 17 indicated R-KHE (Figure 2). After the diagnosis confirmed by the biopsy, the patient was treated with dexamethasone (1 mg/kg, bid) and sirolimus (0.15 mg, bid). After which and in a gradual manner, the patient's hemoglobin and platelet levels became stabilized, and the thoracic and abdominal drainage decreased, with no obvious active bleeding. Fibrinogen fluctuated between 0.6–0.9 g/L during treatment, and gradually rise to normal about 2 weeks after treatment. On April 10, the patient was in a stable condition and was discharged from the hospital with prescriptions of sirolimus and prednisone. The patient was again admitted to the hospital and stayed for chemotherapy with VCR from June 22 to 28. From September 27 to October 26, 2021, the patient was hospitalized again due to intestinal perforation, during which, treatments performed included anti-infection, ileal perforation repair, and abdominal abscess incision and drainage. After discharge, the use of oral drugs was discontinued by the family members without authorization due to their concerns about the side effects of sirolimus and prednisone. The patient was hospitalized from January 16 to 30, 2022 due to severe thrombocytopenia, the patient underwent three times of chemotherapy with VCR, once a week. The abdominal MRI on January 23 was shown in Figure 3. Interventional embolization under the guidance of digital subtraction angiography (DSA) was performed under general anesthesia after comprehensive consideration on January 26. After interventional embolization, platelets of the children gradually return to normal. Oral administrations of sirolimus, prednisone and propranolol were continued for the patient after discharged. The patient received regular outpatient follow-ups. As the platelets gradually stabilized, corticosteroid was gradually reduced within about 5 weeks, and the concentration of sirolimus trough concentration was monitored and maintained at 10–15 ng/ml. Sulfonamide was used to prevent Pneumocystitis Carinii Pneumonia during treatment, and the results of abdominal CT on March 1, 2022 was shown in Figure 4.

### 2.2. Review of the literature

The scope of published literature research was databases including Wanfang, CNKI, and PubMed by March 2022. Key words included Kaposiform hemangioendothelioma (KHE), retroperitoneal Kaposiform hemangioendothelioma (R-KHE), and KMP. After excluding adult cases and those children's cases without full-text articles, a total of 14 cases of R-KHE were retrieved from Chinese and English literatures (6–16).

## 3. Results

### 3.1. Prognosis of the patient

As informed by the follow-up phone calls for 1 year and 2 months, the patient was in good growth and development. The retroperitoneal tumor was significantly smaller as shown by subsequent CT scan, and follow-up tests indicated normal platelet and coagulation functions.

### 3.2. Literature analysis

Together with the case in this report, there are 15 cases of R-KHE in children, as shown in Table 1. The male to female ratio was 12:3, age ranged from 7d to 36 months. Twelve cases underwent biopsy, all 15 cases have clinical symptoms and positive signs, and 14 cases have combined KMP. The main manifestations were abdominal mass in 3 cases, jaundice in 7 cases, abdominal distension and skin purpura in 2 cases, vomiting in 2 cases, and walking instability in 1 case. Auxiliary examinations: 5 cases underwent MRI, and 9 cases underwent abdominal CT. Treatment methods: surgery plus drug therapy for 6 cases, only surgery for 4 cases, only drug therapy for 4 cases, and drug therapy plus radiotherapy for 1 case. Clinical outcomes (Table 2): improvement was observed in 11 cases, with significantly reduced tumor and survival with tumor; complete disappearance of tumor and full clinical recovery in 2 cases; and death in 2 cases.

**Table 1:** General information of R-KHE in children reported in literature review and our case .

S/N	Gender	Age	Location	Clinical presentation	Imaging Manifestation	KMP	Treatment	Follow-up	Outcome
						(Yes/No)			
1 (6)	M	5m	Retroperitoneum	Abdominal mass	Contrast-enhanced CT scan of the abdomen showed a huge mass on the left side of the abdomen, about 7.4 cm × 7.7 cm × 10 cm in size, crossing the midline, with clear borders, and low mixed density with multiple calcifications. The lesion showed obvious inhomogeneous enhancement with contrast.	Yes	Surgery + glucocorticoid + VCR	2y	Tumor disappeared
2 (6)	F	4m	Retroperitoneum	Vomiting, bloody stool	Magnetic resonance imaging (MRI) showed: abnormal mass signal at the root of the mesentery, thickening and edema of the intestinal canal, and a large amount of peritoneal effusion. Computed Tomography Angiography (CTA) showed multiple abnormal enhancement foci in the head of pancreas and root of mesentery, massive effusion in the abdomen and pelvic cavity, thickening of the intestinal wall, edema, and a lot of collateral vessels in the abdominal cavity.	Yes	Surgery + glucocorticoid + VCR	4m	Smaller tumor
3 (7)	M	2y	Retroperitoneum	Abdominal distention, vomiting	A standing plain radiograph of the abdomen indicated a lower intestinal obstruction	No	Surgery	None	Smaller tumor
4 (8)	M	2m	Head of pancreas	Jaundice, petechiae	Abdominal MRI showed a 4.9 cm × 3.8 cm × 3.5 cm mass with unclear borders, retroperitoneal vascular mass infiltrating the head of pancreas and hepatic hilum area, and significantly dilated intrahepatic bile ducts	Yes	Glucocorticoid + sirolimus	2y	Smaller tumor
5 (8)	M	80d	Head of pancreas	Jaundice, purpura	Abdominal MRI showed a 9.9 cm × 5.2 cm × 9.2 cm enhancement, with unclear borders, retroperitoneal vascular tumor infiltrating the head of pancreas and the hepatic portal system	Yes	Glucocorticoid + VCR + sirolimus	2.5y	Smaller tumor
6 (8)	M	73d	Head of pancreas	Jaundice, petechiae	Abdominal MRI revealed a 5.6 cm × 3.2 cm × 2.9 cm vascular mass in the head of the pancreas	Yes	Glucocorticoid + VCR + sirolimus	1y	Smaller tumor
7 (9)	F	8m	Head of pancreas	Jaundice	MRI showed dilated left and right hepatic ducts, common hepatic ducts and common bile ducts, and the head and body of the pancreas were about 4.7 × 5.2 cm, with strong signals. Portal veins and mesenteric vessels are closely connected to the tumor	No	Surgery	28m	Smaller tumor
8 (10)	M	6m	Retroperitoneum	Left-side abdominal mass	CT scan (with contrast) of the abdomen shows a large, vascularized retroperitoneal mass, 6.5 × 5 cm in size	Yes	Surgery + interferon	3y	Smaller tumor
9 (11)	Unknown	8m	Retroperitoneum	Abdominal mass, purpura	CT scan of the abdomen revealed a large polycystic tumor extending from the lower pole of the spleen down to the bladder	Yes	Surgery	18m	Tumor disappeared
10 (12)	M	3y	Retroperitoneum	Unsteady walking	MRI revealed an infiltrating, fatty mass in the left psoas, left quadratus lumborum, and left retroperitoneum.	Yes	Biopsy + sirolimus	1y	Smaller tumor
11 (13)	F	8m	Pancreas	Abdominal distention, jaundice	CT scan showed an enhancing mass in the head of the pancreas, with unclear borders	Yes	Surgery + interferon	None	Death
12 (14)	M	16m	Pancreas	Jaundice, fever	Contrast-enhanced CT scan of the abdomen shows a mass with markedly heterogeneous contents, 2.2 × 1.9 × 2.5 cm in size, located in the head of pancreas	Unknown	Surgery + sirolimus	6m	Smaller tumor
13 (15)	F	7d	Retroperitoneum	Abdominal purpura	Abdominal CT scan indicated large tumor on the left retroperitoneum displacing the kidney downward. With ipsilateral subcutaneous thickening and hemothorax.	Yes	VCR + radiotherapy	None	Smaller tumor
14 (16)	F	28d	Retroperitoneum	Abdominal distention, Jaundice	64-slice CT scans with and without contrast showed a mass of soft tissue shadows in the retroperitoneum, about 5.7 cm × 5.3 cm in size, with obvious enhancement on contrast-enhanced scans, closely related to the celiac trunk, continuous enhancement in the portal venous phase, and necrotic density shadows; intestinal space disorder, with liquid density shadows in the abdominal and pelvic cavities.	Yes	Surgery	None	Death
15	F	36d	Retroperitoneum	Abdominal distention, petechiae	CT scans with and without contrast showed right pleural effusion; right kidney lesions, enlarged right kidney with decreased density, and unclear borders; right kidney perfusion decreased when with contrast, and inhomogeneous enhancement; large areas of obvious enhancement shadow seen in the right perirenal space and retroperitoneal space; peritoneal effusion	Yes	Surgery + glucocorticoid + VCR + Sirolimus	1y	Smaller tumor

M, male; F, female; y, year; m, month; d, day; KMP, Kasabach-Merritt phenomenon; VCR, Vincristine.

**Table 2:** Analysis of treatment methods and follow-up outcomes of 15 cases.

Item	Classification	No. of cases	Percentage
Treatment method	Extensive excision of the lesion	10	67%
	Sirolimus	5	33%
	Dexamethasone	5	33%
	Vincristine	5	33%
	Interferon	5	33%
Outcome	Clinical recovery	2	13%
	Survival with tumor	11	73%
	Death	2	13%

## 4. Discussion

Kaposiform hemangioendothelioma (KHE) is an aggressive tumor that resembles Kaposi's sarcoma (KS) in histology, and most KHE cases manifested in infancy and childhood (4). First reported by Zukerberg in 1993, it was classified as a “locally aggressive or borderline vascular tumor” by the International Society for the Study of Vascular Anomalies (ISSVA) in 2014. According to the morphology of the tumor, Ji et al. (2) divided the tumor into superficial type (limited to the skin and subcutaneous soft tissue, without invading muscle and bone, without invading chest and abdominal cavity), mixed type (involving skin and subcutaneous, deep muscle, bone or joint at the same time) and deep type (involving deep muscle, bone or joint, without skin manifestations). Of which the mixed type was the most common, that's about 63 percent. And only 10% located in the retroperitoneum (4). KHE is one of the main tumors that cause the KMP in infants and young children. Associated with severe (consumption) thrombocytopenia, anemia, secondary fibrinogen reduction and coagulation factor consumption and other clinical presentations, KMP is featured by rapid onset, rapid progression and high mortality (17). R-KHE has diverse clinical presentations and non-specific symptoms and imaging examinations, and often accompanied by KMP. Thus, it has some difficulty in R-KHE diagnosis.

The tumor in our case was located in the retroperitoneum, with severe thrombocytopenia, severe anemia, and coagulation dysfunction when she was admitted to the hospital. Initially we considered it a bleeding disorder caused by some internal disease: immune thrombocytopenia (ITP) or thrombotic thrombocytopenia (TTP)? The patient received glucocorticoid therapy, high-dose gamma globulin pulse therapy, and plasma exchange therapy, but the symptoms showed no improvement. A multidisciplinary consultation was organized, which recommended exploratory laparotomy after infusion of platelets and improvement of coagulation. The subsequent pathological biopsy suggested KHE. Based on this definitive diagnosis, oral administration of sirolimus and dexamethasone was prescribed for the patient.

The incidence of R-KHE is low, and most of the reports are single case study. Both the clinical presentations and auxiliary examinations are non-specific. The diagnosis is mostly based on pathological biopsy, and there are no standard treatment protocols. Most R-KHE in previous reports occurred in the pancreas, and the common clinical presentations include abdominal pain and jaundice. The lesion of our case was in the kidney, with abdominal distension and skin petechiae as the main manifestations. The common auxiliary examinations for R-KHE include abdominal contrast-enhanced CT scan, contrast-enhanced MRI (18), and biopsy. Among them, CT scan is quicker and has better performance in evaluating tumor size and degree of infiltration, but it is insufficient on specificity. MRI is more valuable for diagnosing vascular tumors, but it may be too time-consuming to perform in some critical cases (19). Pathological diagnosis, as the gold standard for the diagnosis of KHE, still has the disadvantage that some patients in critical condition cannot tolerate surgical biopsy. Among all 15 cases, there were 9 cases underwent abdominal contrast-enhanced CT scan, which showed retroperitoneal mass or diffuse low-density shadow, and enhancement when with contrast; 5 cases underwent abdominal contrast-enhanced MRI, with high and low signals on T1-weighted images and high signals on T2-weighted images, and inhomogeneous enhancement after contrast applied; 12 cases underwent surgical biopsy to confirm the diagnosis. It is feasible to perform ultrasound or CT-guided biopsy on KHE patients, which has been reported in previous literature (20). Case 10 was performed by CT-guided puncture biopsy, and the final diagnosis was confirmed. Our case was treated by exploratory laparotomy, because we didn't know the cause of the disease at beginning. Considering the critical status of the patient, we had chosen exploratory laparotomy to stop bleeding, remove hematoma, and make a definite diagnosis.

There are several options for the treatment of KHE, including drug therapy, surgery and arterial embolization. In clinical practice, a combined therapy is often preferred due to the limited efficacy of a single regimen (21). Most cases of KMP occur in infants younger than 6 months, in whom, there may be organ compression as the tumor increasing, accompanied with formation of large hematoma and severe thrombocytopenia. Platelet transfusions can exacerbate this phenomenon and should be avoided if possible. Platelets are given only when the patient has life-threatening bleeding or is preparing for biopsy or tumor resection (21). Most deaths are due to bleeding in vital organs. The mortality could be higher if the tumor is located in the retroperitoneum. Because most R-KHE cases are accompanied by severe thrombocytopenia and coagulation dysfunction, some patients cannot tolerate surgical treatment, but when the patient has life-threatening bleeding, it is proper to consider surgical excision or biopsy after the patient has been treated with platelets and fresh frozen plasma. Among the 15 cases in this study, 10 children received extensive resection of the lesions, which was supplemented by postoperative drug treatment, and 8 of them recovered well after surgery, with rapid recovery of platelet and coagulation functions. The significant reduction in tumor volume may account for the rapid normalization of platelet counts and disappearance of residual lesions. Interventional embolization under the guidance of digital subtraction angiography (DSA) is also an important therapeutic method when the lesions are extensive, unresectable, ineffective in drug therapy, or the coagulation function is not improved and continues to deteriorate (22). Its advantage is that it has a rapid effect. Through embolization of the blood supplying artery, the tumor is ischemia, degeneration, necrosis, shrinkage, and the capture and destruction of platelets in the tumor are reduced (22). In our case, the disease relapsed, and platelets decreased significantly. Interventional embolization was performed to the patient, and platelets gradually returned to normal along with drug therapies.

Despite the multiple drug options for KMP treatment, the reported efficacy varies greatly. Main drugs used in clinical practice include VCR, steroids, sirolimus and propranolol (21). The Expert Consensus on KMP Treatment formulated by Drolet et al. recommended a glucocorticoid + VCR therapy as the initial treatment for KHE patients with KMP (17). Among the cases reported in this study, 6 children were treated with glucocorticoid combined with VCR, and good curative effects were found in all of them. Recent retrospective clinical studies have found that sirolimus has shown good effects in the treatment of KHE, even in patients that being resistant to steroid, combined with KMP and showing no response to VCR, and the effects are long-lasting (23). The six children received oral sirolimus treatment, and the treatment achieved good effects. The dosage is: sirolimus [0.08 mg/kg, orally, twice a day, to maintain the plasma drug concentration at 10–15 ng/ml]. In most cases, the drug takes effect in 5 to 10 days, and the drug concentration in plasma reaches a stable level in 3 to 4 weeks (23). If the drug treatment is effective, the platelet level will increase significantly in a short period of time, the coagulation function will also be significantly improved; and in some patients bloody pleural effusion or peritoneal effusion will also be reduced. Recent studies (24) have shown that short-term prednisolone combined with sirolimus is superior to sirolimus monotherapy in improving the signs and symptoms of KMP-active KHE. In our case, sirolimus combined with prednisone was treated orally after discharge, and the patient gradually decreased and stopped prednisone treatment about 5 weeks after platelet stabilization during follow-up. However, improper feeding method, severe vomiting and jaundice will affect the absorption and effectiveness of sirolimus, so close monitoring of its plasmatic concentration is the key to achieving good therapeutic effects (25). As reported in literature, the short-term toxic side effects of sirolimus mainly include immunosuppression, elevated transaminases, hyperlipidemia, mucositis, and delayed vaccination (25). The patient in this study developed intestinal perforation and complications of intestinal infection 6 months after oral sirolimus treatment, but improved after active anti-infection and surgical treatment. Therefore, we need to monitor closely during treatment and be alert to the side effects of sirolimus. In our case, family members of the patient stopped sirolimus by themselves, which resulted in relapse. Therefore, it is important to strengthen the education of the family members in the process of diagnosis and treatment.

Together with the cases in the literature and our study, we conclude the following revelations: (1) R-KHE occurs in very young patients, mostly in infants of 1 to 4 months old, and R-KHE is often accompanied with KMP; (2) R-KHE shall be distinguished from solid retroperitoneal tumors and hematological tumors, such as idiopathic thrombocytopenic purpura; (3) the clinical presentations of R-KHE are diverse, mainly including abdominal distension, abdominal mass, jaundice, skin petechiae and ecchymoses. The symptoms are nonspecific; (4) Imaging manifestations of R-KHE: CT scan without contrast showed mass or diffuse low-density shadows in the retroperitoneum, and the lesions were obviously enhanced when with contrast, and some were accompanied by peritoneal effusion. Contrast-enhanced MRI may also be used to assist diagnosis; (5) for patients with above symptoms and imaging manifestations, if with accompanied KMP and the condition is critical, surgical biopsy can be performed after improvement of coagulation and infusion of platelets. If biopsy is not feasible due to the condition, clinical judgement could be made based on previous diagnosis and treatment experience, and diagnostic treatment with drugs could be performed; (6) if the effect of single drug therapy is unsatisfactory, a combination therapy could be considered.

## 5. Conclusion

The clinical presentations of R-KHE are diverse and non-specific, most of which are associated with KMP, with rapid onset, rapid progression, and high mortality. Therefore, we should be wary of this disease in children with severe thrombocytopenia, anemia, severe coagulation dysfunction combined with intra-abdominal hemorrhage. Intervention-guided needle biopsy can be performed, It can also be treated with surgery if the condition permits. But, if the patient is in a critical condition and cannot tolerate surgery, a comprehensive judgment shall be made with combined consideration of the medical history, and diagnostic treatment with drugs could be performed. At present, sirolimus shows good outcomes in KHE treatment, but the plasmatic concentration should be monitored during medication, and attention should be paid to the side effects. If the treatment effect is not good, a combination with other drugs should be considered.

## Data Availability

The raw data supporting the conclusions of this article will be made available by the authors, without undue reservation.
